# Resistance against Membrane-Inserting MmpL3 Inhibitor through Upregulation of MmpL5 in Mycobacterium tuberculosis

**DOI:** 10.1128/AAC.01100-20

**Published:** 2020-11-17

**Authors:** Ming Li, Samuel Agyei Nyantakyi, Mei-Lin Go, Thomas Dick

**Affiliations:** a Department of Medicine, Yong Loo Lin School of Medicine, National University of Singapore, Singapore; b Department of Pharmacy, Faculty of Science, National University of Singapore, Singapore; c Department of Microbiology and Immunology, Yong Loo Lin School of Medicine, National University of Singapore, Singapore; d Center for Discovery and Innovation, Hackensack Meridian Health, Nutley, New Jersey, USA; e Department of Medical Sciences, Hackensack Meridian School of Medicine, Nutley, New Jersey, USA; f Department of Microbiology and Immunology, Georgetown University, Washington, DC, USA

**Keywords:** MmpL3, MmpR5, MmpL5, indolyl Mannich bases

## Abstract

Spiroketal indolyl Mannich bases (SIMBs) present a novel class of membrane-inserting antimycobacterials with efficacy in a tuberculosis mouse model. SIMBs exert their antibacterial activity by two mechanisms. The indolyl Mannich base scaffold causes permeabilization of bacteria, and the spiroketal moiety contributes to inhibition of the mycolic acid transporter MmpL3. Here, we show that low-level resistance to SIMBs arises by mutations in the transcriptional repressor MmpR5, resulting in upregulation of the efflux pump MmpL5.

## TEXT

The membrane is an attractive but underexplored target in the discovery of novel antimycobacterials (1, 2). Amphiphilic indolyl Mannich bases were shown to insert into and permeabilize the mycobacterial membrane, thus killing both growing and nongrowing bacilli (3). Consistent with their membrane-disrupting mechanism of action, resistance mutants could not be isolated (3). Incorporation of a spiroketal moiety in the Mannich base caused a 10-fold increase in potency (4). Interestingly, mutants resistant to the spiroketal analogs could be isolated and mapped to the mycolic acid transporter MmpL3 (5). Biochemical, metabolic, computational, and structure-activity relationship analyses revealed that the potency improvement was caused by the acquisition of a second mechanism of action due to the inclusion of the spiroketal moiety (5). In addition to permeabilizing the membrane, spiroketal analogs of the indolyl Mannich bases (SIMBs) inhibit the flippase activity of the transmembrane MmpL3 protein and, hence, the transport of mycolic acids from the cytoplasm to the periplasmic space (5). Thus, SIMBs are novel dual-mechanism antibacterials, disrupting the integrity of the bacterial cell membrane and blocking the transport of an essential cell wall component by inhibiting a transmembrane transporter (5). Consistent with this dual mechanism, missense mutations at the binding site of SIMBs on MmpL3 reverted the 10-fold potency increase achieved by the addition of the spiroketal moiety (MIC_90_ = 1 μM) back to that observed for nonspiroketal Mannich bases (MIC_90_ = ∼10 μM), which act only by disrupting membrane integrity (5). Thus, the membrane-permeabilizing mechanism endowed by the amphiphilic indolyl Mannich base scaffold of SIMBs ensures that these compounds retain appreciable activity even after bacteria have acquired resistance to the second, MmpL3-related mechanism (5). Importantly, the lead compound of these dual-mechanism SIMBs, termed SIMB lead or SIMBL (9-[(6-methoxy-1-octyl-1*H*-indol-3-yl)methyl]-1,5-dioxa-9-azaspiro[5.5]undecane), showed efficacy in a mouse model of tuberculosis, hence providing *in vivo* proof of concept for this novel approach (4). Taken together, prior work identified SIMBL as a promising lead antimycobacterial with a novel dual mechanism of action, bactericidal activity against growing and nongrowing drug-tolerant bacilli, and efficacy in a tuberculosis mouse model (3–5).

In this study, we asked whether genetic resistance to SIMBL may also emerge via non-MmpL3-related indirect mechanisms. In the previous target deconvolution work, we employed high concentrations (4× and 8× MIC_90_; broth MIC_90_ = 1 μM) of SIMBL for the selection of spontaneous resistance mutants, delivering exclusively on-target missense mutations in MmpL3 with a frequency of 10^−8^/CFU (5). To identify additional, lower-level, off-target mechanisms of resistance to SIMBL, we repeated mutant selection with Mycobacterium tuberculosis H37Rv (ATCC 27294) as described in reference 5 but on Middlebrook 7H10 agar containing a lower concentration (3× MIC_90_; 3 μM) of SIMBL. Plating of 5 × 10^8^ bacteria resulted in four resistant colonies that were restreaked on SIMBL-containing agar for confirmation of resistance and colony purification. Discrete colonies were then cultured, and MIC_90_ values of SIMBL in Middlebrook 7H9 broth were determined as described (6). Two strains showed a 10-fold increase in MIC_90_, and two strains showed a 4-fold increase in MIC_90_. Targeted Sanger sequencing of *mmpL3* revealed that the two higher-level resistance mutants harbored missense mutations in *mmpL3*, T959C/L320P and G1772T/S591I, as reported previously (5), whereas the two lower-level resistance strains M1 and M2 carried wild-type alleles of *mmpL3* (Table 1). Mutant selection was also performed with Mycobacterium bovis BCG (ATCC 35734). A total of 10^8^ bacteria were plated on agar containing SIMBL at 2× MIC_90_ (2 μM) resulting in one strain, B1, with a 4-fold increased MIC_90_ (Table 1). Sequencing of *mmpL3* in B1 also revealed a wild-type allele. To determine the mechanism underlying this low-level resistance not associated with MmpL3, the two M. tuberculosis strains M1 and M2 and the M. bovis BCG strain B1 were subjected to whole-genome sequencing as described previously (5, 7). All three strains harbored mutations in *mmpR5* encoding a nonessential transcriptional repressor (Table 1) (8, 9). The polymorphisms identified in *mmpR5* were verified by targeted Sanger sequencing using the reported primers 5′-GCACGCTTGAGAGTTCC-3′ and 5′-CGCCGTCTTGCTCGC-3′ (10). Two resistant strains showed missense mutations in the DNA-binding domain (A202G/S68G in M1) and the dimerization domain (G73T/G25C in M2) of MmpR5, respectively (Table 1) (8). The third strain showed a frameshift mutation (Ins68T in B1) in the N-terminal part of MmpR5, leading to a truncated product devoid of both domains (Table 1) (8). The nature and location of the observed resistance mutations in the MmpR5 protein suggest that they may affect its function as a DNA-binding repressor.

**TABLE 1:** MIC_90_ of SIMBL for wild-type and SIMBL-resistant M. tuberculosis and M. bovis BCG strains and polymorphisms in *mmpR5*

Strain	MIC_90_ (μM)^*a*^	Mutation	
		*mmpR5*^*b*^	Other genes^*c*^
M. tuberculosis wild type	1.0		
M. tuberculosis M1	3.9	A202G/S68G	*Rv0907*, C1190A/T397K
M. tuberculosis M2	4.0	G73T/G25C	
M. bovis BCG wild type	1.0		
M. bovis B1	4.2	Ins68T/truncation	*BCG_2955*, Ins2684C/truncation

a MIC_90_ is the concentration of SIMBL required to inhibit 90% of bacterial growth in broth culture compared to an untreated drug-free control. Means of three independent determinations are shown. Synthesis of the spiroketal indolyl Mannich base lead compound SIMBL (9-[(6-methoxy-1-octyl-1*H*-indol-3-yl)methyl]-1,5-dioxa-9-azaspiro[5.5]undecane) was described previously (4).

b *mmpR5*, *Rv0678* in M. tuberculosis and *BCG_0727* in M. bovis BCG.

c Polymorphisms in other genes detected by whole-genome sequencing.

MmpR5 was reported to repress expression of its neighboring, divergently transcribed siderophore transporter and multisubstrate efflux pump gene *mmpL5* (mycobacterial membrane protein large 5) and is hence named MmpR5 (mycobacterial membrane protein repressor 5) (10–16). Notably, numerous MmpR5 mutations have been associated with mycobacterial resistance to a range of chemically and mechanistically diverse drugs, including azoles, bedaquiline, clofazimine, the ionophores nigericin and A23187 (calcimycin), thiacetazone, and imidazo[1,2-*b*][1,2,4,5]tetrazine derivatives (10, 16–28). In fact, the SIMBL resistance mutation in the DNA-binding domain of MmpR5 detected in M. tuberculosis M1 (A202G/S68G) is known to confer resistance to bedaquiline and clofazimine (18, 19). Resistance-conferring mutations in MmpR5 disable its transcriptional repressor function, resulting in overexpression of the MmpL5 pump and increased expulsion of drugs (10, 16–28). Consistent with this model, cotreatment of MmpR5 mutants overexpressing MmpL5 with drugs and the efflux pump inhibitor reserpine reverted resistance to bedaquiline (18).

We hypothesized that a similar MmpL5-mediated mechanism of resistance may also underly the 4-fold resistance of mycobacteria to SIMBL. To examine this hypothesis, we first tested the prediction that SIMBL resistance due to MmpR5 mutations should be phenotypically reverted by the efflux pump inhibitor reserpine as observed for bedaquiline (18). We cotreated the SIMBL-resistant strains M1, M2, and B1 with SIMBL and reserpine and observed that reserpine indeed restored wild-type susceptibility of all three MmpR5 mutant strains (Table 2).

**TABLE 2:** MIC_90_ of SIMBL, bedaquiline, and isoniazid for wild type and SIMBL-resistant M. tuberculosis and M. bovis BCG strains without or with reserpine^*a*^^,^^*b*^

Compounds	MIC_90_ (μM)				
	M. tuberculosis			M. bovis BCG	
	Wild type	M1	M2	Wild type	B1
Reserpine	>100	>100	>100	>100	>100
SIMBL	1.0	3.9	4.0	1.0	4.2
SIMBL + reserpine	0.6	0.6	0.6	0.5	0.5
BDQ	0.8	5.0	4.8	0.12	1.0
BDQ + reserpine^*c*^	0.08	0.15	0.15	0.02	0.04
INH	3.2	3.2	3.2	3.2	3.2
INH + reserpine	3.2	3.2	3.2	3.2	3.2

a MIC_90_ is the concentration of drug required to inhibit 90% of bacterial growth in broth culture compared to an untreated drug-free control. Means of three independent determinations are shown. SIMBL, 9-[(6-methoxy-1-octyl-1*H*-indol-3-yl)methyl]-1,5-dioxa-9-azaspiro[5.5]undecane; BDQ, bedaquiline; INH, isoniazid. SIMBL was synthesized as described (4), other drugs were purchased from Sigma-Aldrich. Drug solutions were prepared in 100% dimethyl sulfoxide, except for reserpine, which was dissolved in deionized water.

b Efflux pump inhibitor reserpine was added at a subinhibitory concentration of 25 μM.

c As described previously, a potentiating effect of reserpine on the activity of bedaquiline was observed for wild-type bacteria (18).

Next, we tested the prediction that M1, M2, and B1 should display cross-resistance to other drugs subject to the MmpR5-MmpL5 resistance mechanism and chose bedaquiline as our test compound (16, 18–25). The MIC_90_ of bedaquiline against all three mutants was 6- to 8-fold higher compared to the wild type (Table 2), thus demonstrating cross-resistance. Consistent with previous reports, resistance to bedaquiline was also phenotypically reversable by cotreatment with reserpine (Table 2) (18). In contrast, susceptibility to isoniazid was not altered in SIMBL/bedaquiline-resistant M1, M2, and B1 strains (Table 2) (16), suggesting that the observed effects are drug specific and not due to general drug resistance caused by MmpR5 mutations.

**FIG 1:**
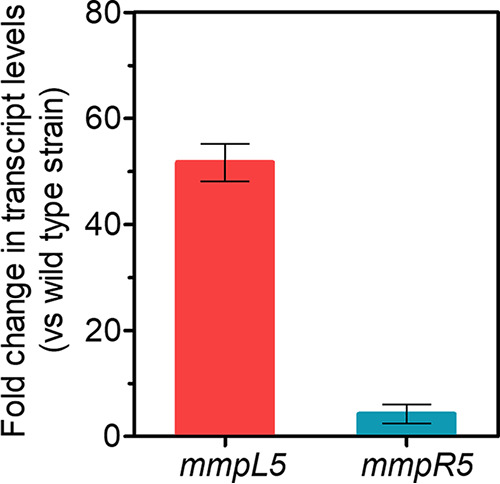
Effect of SIMBL resistance mutations in MmpR5 on *mmpL5* mRNA level. Fold change in transcript level of *mmpL5* in SIMBL-resistant M. bovis BCG B1 strain compared to that of the wild-type strain is shown. Transcript levels were measured by quantitative reverse transcription-PCR analysis and normalized against the internal invariant control 16S rRNA (29, 30). Mean values and standard deviations from triplicate determinations are shown. Consistent with previous reports, mutations in MmpR5 also resulted in upregulation of *mmpR5* itself due to the gene’s autoregulation (10, 16, 28). Primers used in quantitative PCR were 5′-ATGACGGCCTTCGGGTTGTAA-3′ and 5′-CGGCTGCTGGCACGTAGTTG-3′ for 16S rRNA, 5′-GACCAACCTGCTCGTG-3′ and 5′-CGCCGAACATGGTGTA-3′ for *mmpL5*, and 5′-AATGCCCGGATGCTGAT-3′ and 5′-CTGCAGTTCGGCCATTG-3′ for *mmpR5* (10, 30).

Finally, we tested the prediction that mutations in MmpR5 should increase the transcript level of the efflux pump gene *mmpL5*. Total RNA was extracted from M. bovis BCG B1 and subjected to quantitative reverse transcription-PCR analysis using 16S rRNA as the internal invariant control as described (29, 30). Compared to wild-type M. bovis BCG, B1 showed a more than 50-fold increase in *mmpL5* mRNA level (Fig. 1), suggesting derepression of the pump gene *mmpL5* in the MmpR5 mutant background.

In conclusion, we report the identification of a pump-based resistance mechanism to the spiroketal indolyl Mannich base lead SIMBL (9-[(6-methoxy-1-octyl-1*H*-indol-3-yl)methyl]-1,5-dioxa-9-azaspiro[5.5]undecane). This resistance mechanism arises from mutations in the transcriptional repressor MmpR5, resulting in the overexpression of the efflux pump MmpL5. MmpL5-mediated resistance has been reported for multiple antimycobacterials (10, 16–28). Thus, our finding adds SIMBL to the growing list of putative substrates of the MmpL5 efflux pump. SIMBL is the first membrane-anchored agent and the first MmpL3 binding inhibitor subject to this pump-based resistance mechanism in M. tuberculosis.
